# Associative emotional memory encoding: insights from network stability analysis of an fMRI-driven bilinear dynamics

**DOI:** 10.3389/fnsys.2026.1794170

**Published:** 2026-06-08

**Authors:** Weronika Dziarnowska, Melis Orhun, Yannan Zhu, Nils Kohn, Guillén Fernández, Matin Jafarian

**Affiliations:** 1 Delft University of Technology, Delft, Netherlands; 2 Donders Institute for Brain, Cognition and Behaviour, Radboud University Medical Center, Nijmegen, Netherlands

**Keywords:** dynamic causal modeling, emotional associative memory, fMRI, network analysis, systems and control theory

## Abstract

**Objectives:**

The interplay between emotion and memory is a central topic in cognitive neuroscience, with open questions about the underlying neuronal mechanisms. This article aims to study the effects of order and intensity of emotional information on associative memory encoding. To this aim, we employ dynamic causal modeling to model the dynamic network composed of the hippocampus, amygdala, and orbitofrontal cortex during an fMRI associative memory encoding task and apply graph and control theory tools to obtain novel insights.

**Methods:**

Participants were clustered into three condition groups, neutral–neutral, neutral–emotional, or emotional–emotional, and viewed image pairs associated with their assigned condition. Using the dynamic causal modeling framework, we explore several dynamic models and show that a stochastic bilinear state-space model best describes the neuronal dynamics in all conditions. Furthermore, we use graph and control theory techniques to both validate and analyze the model. Particularly, we analyze the network dynamics of each condition using tools from graph theory and stability theory and discuss the differences in the strength and direction of connectivity as well as stability of each of these networks.

**Results:**

We confirm the prior finding that memory is enhanced in the neutral-emotional condition. In our work, this enhanced memory is associated with the increased hippocampus–amygdala coupling strength in this condition. Moreover, we show that in the emotional–emotional condition, coupling of hippocampus and amygdala, as well as the whole network connectivity increases. We further predict that the hippocampus–amygdala connectivity in this condition increases, when the first image's valence is substantially less negative rated than the second image, but decreases otherwise. This pattern mirrors the neutral–emotional condition, where the first image is emotionally neutral compared with the second. Moreover, our model-based analyses suggest that the amygdala predominantly influences the other two regions in the neutral–emotional condition.

**Conclusion:**

Combined data-driven DCM modeling, stability analyses, and graph-theory tools led to new insights and enhanced the mechanistic understanding of dynamics of emotional associative memory. We discuss these insights, utilize these analytical tools to generalize our findings to some unmeasured conditions, and highlight the potential of these techniques to inform the development of future technological or pharmacological approaches targeting regulatory mechanisms.

## 1 Introduction

The intricate relationship between emotions and memory has long fascinated researchers due to its profound implications for understanding human cognition and behavior, central to cognitive neuroscience and psychiatric research (Ochsner and Schacter, 2000). Emotional experiences are often remembered more vividly than neutral ones, shaping how individuals perceive and interact with the world (Cahill and McGaugh, 1996; LaBar and Cabeza, 2006). Yet, the neural mechanisms that govern emotional associative memory remain partially understood.

The brain regions involved in memory-emotion interactions include the amygdala, the hippocampus, and specific prefrontal areas. The amygdala is mainly involved in assigning emotional value to sensory stimuli and in modulating memory consolidation and retrieval (MorÉn, 2001) while the hippocampus supports the formation and retrieval of declarative, associative memory (Eichenbaum, 2004). The orbito-frontal cortex (OFC) within the PFC exerts top-down control by integrating emotion regulation, attention, working memory, and reward processing to support flexible, goal-directed behavior (Miller and Cohen, 2001). To reveal the mechanisms of brain networks in cognition, data collected by functional magnetic resonance imaging (fMRI) has often been used. The technique offers non-invasive high-resolution imaging capable of capturing all brain regions, their structures, connectivity, and activity. Although some interactive mechanisms have been delineated, a comprehensive understanding has not yet been achieved.

To identify and predict underlying neural dynamics, several approaches have been proposed in the literature, including generative models, which are typically formulated as systems of differential equations or density dynamics and are often represented within a state-space framework (Ramezanian-Panahi et al., 2022). These models enable the study of dynamic interactions, support mechanistic interpretations of observed data, and provide a foundation for brain simulation and stimulation. Generative models vary in their level of abstraction, the extent of biological detail incorporated, the amount of prior knowledge required, and the size of the datasets they can accommodate (Bakels et al., 2025; Jafarian et al., 2023).

Detailed biophysical models require substantial prior knowledge and involve the estimation of a large number of parameters, specially for large scale neural dynamics, often resulting in considerable complexity in capturing underlying biological processes (Izhikevich and Edelman, 2008). This complexity can hinder parameter estimation, analysis, and inference. While purely data-driven methods minimize reliance on prior biological knowledge (Chen et al., 2018; Deco et al., 2017), hypothesis-driven models lie between detailed biophysical and purely data-driven approaches. These methods, such as Dynamic Causal Modeling (DCM), aim to estimate both the parameters of partially known biophysical models as well as the remaining model structure.

Dynamic Causal Modeling, in particular, is a widely used framework for modeling effective connectivity, that is, the causal influence that neuronal systems exert on each other (Friston et al., 2003). It describes the temporal evolution of latent neuronal states and associated hemodynamic responses under prior assumptions, enabling the estimation of endogenous, modulatory, and driving influences within a specified network. DCM has been extensively applied to investigate the effective connectivity in brain networks (Daunizeau et al., 2011; Lohmann et al., 2012; Friston et al., 2013; Lohmann et al., 2013), with extensions incorporating stochastic dynamics (Li et al., 2011), excitatory and inhibitory populations (Marreiros et al., 2008) and nonlinear formulations (Stephan et al., 2008).

Prior work has utilized DCM to study emotional memory mainly focusing on the amygdala and hippocampus interactions. Using deterministic two-state bilinear DCM, where each brain region is modeled with two interacting neural states (typically excitatory and inhibitory), and inter-regional couplings are modulated by external inputs, Fastenrath et al. (2014) reported that the strength of the connection from the amygdala to the hippocampus was rapidly and robustly increased during the encoding of emotional pictures compared to the neutral ones. Another fMRI study employed classical DCM, i.e., inter-regional couplings are modulated by external inputs, to investigate the effective connectivity among the amygdala, hippocampus, and dorsolateral prefrontal cortex during a memory–emotion task (Gagnepain et al., 2017). Their analysis showed that the suppression of distressing memories requires PFC regions to inhibit both amygdala and hippocampus activities. Using classical DCM, a further study on emotional associative memory retrieval demonstrated that the OFC modulates amygdala-hippocampus interactions during the recall of emotional context (Smith et al., 2006).

These studies demonstrate the utility of DCM in revealing effective connectivity in brain regions involved in emotional memory. To date, to the best of our knowledge, the obtained models have been mainly used for inferring effective connectivity and not for qualitative analysis of the network behavior. In fact, differential equations representing the emotional memory dynamics are capable of providing insights on the whole network performance and can be used for prediction and regulation. In this work, we aim at addressing this gap by modeling and analyzing the emotional memory encoding network in a task where the order and valence of negative emotions matter.

The recent study in Zhu et al. (2023) has performed fMRI data analysis from an experiment involving the memorization of neutral and emotionally charged image pairs and explored how emotional stimuli influence memory integration. Participants were clustered to three condition groups and viewed image pairs that were either neutral–neutral, or neutral–emotional, or emotional–emotional. The study has used task-dependent functional connectivity that allows measuring correlated activities among brain regions. Their findings suggested that emotional information facilitated memory integration with related neutral information but disrupted the integration with other emotional information.

In this work, we develop a dynamic model of the emotional associative memory encoding task reported in Zhu et al. (2023). Our aims are to: (1) use Dynamic Causal Modeling (DCM) to derive a set of differential equations, i.e., dynamic model, that reproduce the data and captures the underling neuronal dynamics of emotional associative memory; (2) validate the dynamical properties of the model through stability and controllability analyses; (3) infer effective connectivity among the amygdala, hippocampus, and orbitofrontal cortex (OFC); (4) apply graph-theoretic measures to assess network connectivity; and (5) evaluate how emotional valence, as the model input, affects the model's dynamic properties, particularly stability. We expect the model to generalize to some unmeasured conditions. We discuss the results, limitations, and directions for future research.

## 2 Materials and methods

This section details the experimental paradigm, data acquisition and processing procedures, modeling framework and the model space employed to obtain the associative memory encoding dynamics.

### 2.1 Experimental paradigm

The data used in this research have been derived from the associative encoding phase of a functional magnetic resonance imaging (fMRI) experiment designed to investigate how emotional information influences associative memory (Zhu et al., 2023). During this phase, participants were shown 48 different “ABC” image triplets, where a single spatial location cue (A) was paired first with one image (B), and then the same location was paired with a second image (C), forming AB and AC pairs. The images B and C carried emotional valence; either neutral or negative. Participants were instructed to vividly imagine the relationship between the location and the associated image to facilitate memory formation.

A total of 70 healthy young adults completed the experiment. They were randomly assigned to one of three condition groups, based on the emotional valence of the image stimuli they were exposed to:

- **Neutral–Neutral (NN) group with 25 participants:** both associated images (B and C) in a triplet were neutral,
- **Neutral–Emotional (NE) group with 21 participants:** the first image (B) was neutral and the second image (C) was emotional (negative),
- **Emotional–Emotional (EE) group with 24 participants:** both images (B and C) were emotional (negative).

**Figure 1:**
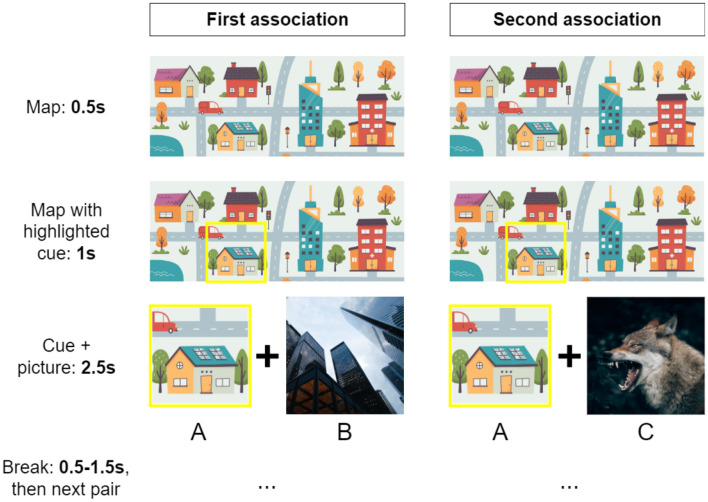
Illustration of the associative encoding trial paradigm used in the experiment. Adapted from Zhu et al. (2023).

The 48 ABC triplets were split into four sets of 12 triplets. During the study, each participant completed four runs of the experiment, with a different set of 12 used per run. In each run, the AB and AC pairings were displayed in a blocked and repeated manner: 12 AB pairs were shown in consecutive encoding trials (explained below), then 12 AC pairs, followed by a repetition of the same AB and AC pairs, concluding a run. Throughout the experiment, participants were scanned using fMRI, and their blood-oxygen-level-dependent (BOLD) responses were recorded. As shown in Figure 1, each encoding trial (AB or AC) consisted of:

1. A brief display of a cartoon map (0.5 s),
2. A highlighted location on that map (1.0 s), and
3. A simultaneous display of the location cue (A) and the associated item (B or C) (2.5 s).

Trials were separated by a jittered inter-trial interval ranging from 0.5 to 1.5 seconds in which a fixation cross was displayed. As a result, each trial lasted approximately 5 seconds and each run around 4 minutes and 30 seconds. The experiment procedure also included recall and behavioral analysis phases. However, this study focuses exclusively on the encoding phase, as it provides the most direct insight into the neural mechanisms of emotional associative memory formation.

#### 2.1.1 Data acquisition

MRI data were acquired using a 3.0 T Siemens Skyra (Siemens Medical, Erlangen, Germany) with a 32-channel head coil system at the Donders Institute, Centre for Cognitive Neuroimaging in Nijmegen, the Netherlands. Functional images were collected using a multi-band echo-planar imaging (mb-EPI) sequence (slices, 66; multi-slice mode, interleaved; slice thickness, 2 mm; TR, 1,000 ms; TE, 35.2 ms; flip angle, 60°; multiband accelerate factor, 6; voxel size, 2 × 2 × 2 mm; FOV, 213 × 213 mm). To correct for spatial distortions, fieldmap images were acquired (slices, 66; multi-slice mode, interleaved; slice thickness, 2 mm; TR, 500 ms; TE1, 2.80 ms; TE2, 5.26 ms; flip angle, 60°; voxel size, 2 × 2 × 2 mm; FOV, 213 × 213 mm). Structural images were acquired using a three-dimensional sagittal T1-weighted magnetization-prepared rapid gradient echo (MPRAGE) sequence (slices, 192; slice thickness, 1 mm; TR, 2300 ms; TE, 3.03 ms; flip angle, 8°; voxel size, 1 × 1 × 1 mm; FOV, 256 × 256 mm).

### 2.2 ROIs and DCM data processing

This section describes the data processing pipeline for DCM. For preliminary processing of fMRI images, we refer to (Zhu et al., 2023). The present study focuses on region-of-interest (ROI) including the amygdala (Amy), hippocampus (Hip) and orbitofrontal cortex (OFC), restricted to the left hemisphere. The selection of ROIs was guided by prior work (Zhu et al., 2023) using the same dataset, which focused on the hippocampus and the left amygdala. In line with our hypothesis-driven modeling approach, we therefore restricted the analysis to left-lateralized ROIs. We further reflect on this choice in Section 4. These anatomical regions are identified using the Automated Anatomical Labeling (AAL) atlas via the WFU_PickAtlas toolbox in MATLAB, which generates binary brain masks.

The fMRI dataset consists of voxels, units on a 3D grid, in the brain regions and a corresponding time series for each voxel. The goal of DCM data processing is to identify the task-relevant voxels within specific brain regions (Volumes of Interest, VOIs), and then average their time series to obtain a single representative signal per VOI. Task-relevant voxels are identified by fitting a Generalized Linear Model (GLM) to the BOLD signal at each voxel. A voxel is considered significant if its activity correlates with the experimental design, here, showing significant responses to both inputs. The GLM is defined as Ashburner et al. (2014):


$$\begin{array}{l} y_{voxel}\left(t\right)=X\beta +\epsilon \end{array}$$

where *y*_*voxel*_(*t*) is the voxel's observed BOLD signal over time, *X* is the design matrix, i.e., input signal convolved with a Hemodynamic Response Function(HRF), β are the parameter estimates, ϵ is the residual noise. The procedure for reducing voxel-level data to a single time series per VOI is provided in the Supplementary material. For DCM data processing, we have used the Statistical Parametric Mapping (SPM12) software package running in MATLAB (Ashburner et al., 2014). At the end of pre-processing, participants with no significant voxels in at least one VOI were excluded leaving a final sample of 65 participants. To obtain DCM models, we used the data corresponding to first of four experimental runs per participants in Zhu et al. (2023), allowing us to focus on neural dynamics during the early stages of learning, before participants develop strategies across repeated exposures. We also note that the number of participants in each condition is sufficient for reliable group-level modeling. Moreover, the first 10 initial values from each run has been discarded due to the scanner setup.

The timing parameters for fitting data to DCM models were chosen to correspond to the experimental conditions. The echo time was to 40*ms* matching the property of the fMRI machine used (Zhu et al., 2023). In addition, slice-timing correction was applied to compensate for the delays within the Temporal Resolution (TR) of 1 sec. The time series were realigned to a reference time at the TR midpoint, following SPM12 recommendations.

### 2.3 Modeling framework

As motivated in the introduction, we chose a DCM framework for developing a state-space model that captures the dynamics of a three-region network comprising the Amygdala (Amy), Hippocampus (Hip), and Orbitofrontal Cortex (OFC). To this aim, we need to choose a model structure, e.g. bilinear, nonlinear, etc, external input signals, and the assumed connection across the network nodes, i.e., brain regions, as well as the manner that the exogenous inputs affect the connections or region dynamics. DCM estimates effective connectivity among brain regions using variational Bayesian inference under free-energy principle (Friston et al., 2003), briefly, a unified theory of how the brain combines prior knowledge with stimuli from the environment to learn and adapt.

**Figure 2:**
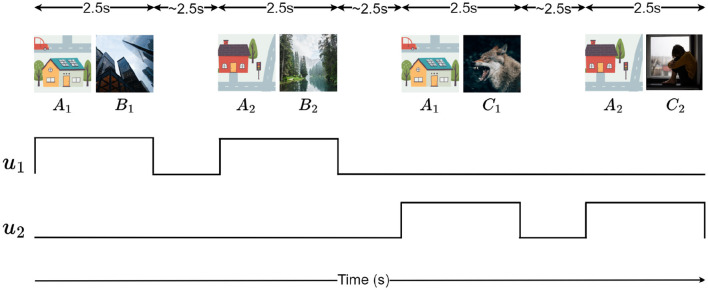
Example of input signals *u*_1_ and *u*_2_ used for fitting DCM in a dummy case with only two triplets in condition NE.

The models' outputs are the averaged time series per VOI obtained after the processing steps explained in Section 2.2. The inputs to our models are the deterministic signals defined as a step function takes the value 1 during external stimulation and 0 otherwise. As described in Section 2.1, each encoding trial has three stages: map, location cue, and simultaneous display of the item pair AB or AC. Because associative encoding is expected to occur only during co-presentation of AB or AC, the inputs are set to 1 only for this 2.5*s* and to 0 during the map, cue, and the jittered inter-trial interval. To distinguish first (AB) and second (AC) associations within each triplet, two separate input signals are defined: *u*_1_ for AB and *u*_2_ for AC. These functions are illustrated in Figure 2.

Models are identified using SPM12 on MATLAB. The algorithm expects the user to define which model connections are assumed. These connections are usually defined by using biological assumptions. If a connection is assumed, its value will be updated during model fitting, otherwise it will remain 0. In what follows, we provide a review on model structures, classic and stochastic DCM. An overview on nonlinear and two-state DCM, used in model comparison, is provided in Supplementary material.

#### 2.3.1 Classical (Bilinear) DCM

**Figure 3:**
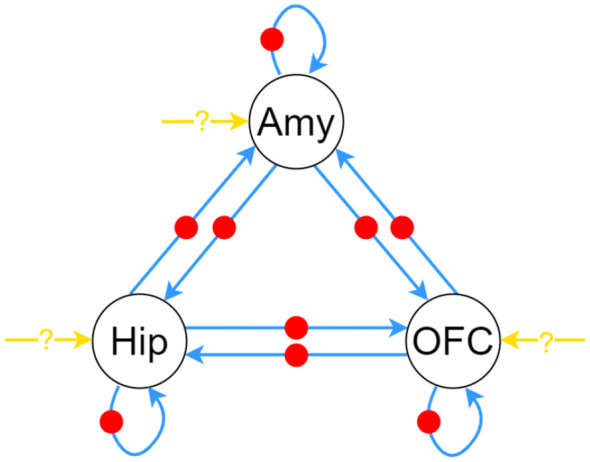
Model space for classical DCM. Blue arrows show couplings between nodes (*A*); dots show input-dependent modulation of couplings (*B*_*i*_); yellow arrow with question mark indicates direct driving effects of the external input on node states (*B*).

DCM modeling is composed of two dynamics: neuronal and hemodynamic states. For neuronal dynamics both model structure, inputs and parameters need to be identified, whereas the identification of hemodynamic only requires parameter identification. The neuronal dynamics are expressed by a nonlinear function that can be approximated by the Taylor Series expansion. Only the first-order derivatives are included in the classical DCM. The neuronal dynamics representing the Classical (Bilinear) DCM are:


$$\begin{array}{l} ż=F\left(z,u,\alpha \right)\\ ż\approx Az+\sum u_{i}B_{i}z+Bu\\ \;=\left(A+\sum u_{i}B_{i}\right)z+Bu\\ A=\frac{\partial F}{\partial z}=\frac{\partial ż}{\partial z},\;B=\frac{\partial F}{\partial u},\;B_{i}=\frac{\partial^{2}F}{\partial z\partial u_{i}}=\frac{\partial }{\partial u_{i}}\frac{\partial ż}{\partial z}, \end{array}$$

with *z* as states, *u* as inputs and α as the parameters of the model. Matrix *A* represents anatomical connections between the brain regions, *B*_*i*_ the change in coupling caused by the j-th input and *B* the direct influence of inputs on neuronal dynamics. Figure 3 shows the structure of the model.

In addition to these dynamics, the DCM framework uses the Balloon model (Buxton et al., 1998; Mandeville et al., 1999; Buxton and Frank, 1997) for the hemodynamic which includes vasodilator signals, inflow, blood volume and normalized deoxyhemoglobin content. The dynamics explains how the activity of neural regions influence hemodynamic responses. When combined with the neuronal dynamics, the full model of the system is obtained. The final model then contains *x* as the states of both models, *u* the inputs and θ all of the parameters to be estimated for both models:


$$\begin{array}{l} ẋ=f\left(x,u,\theta \right)\\ y=h\left(u,\theta \right)+X\beta +\varepsilon , \end{array}$$

where *y* is the output, *h*(*u*, θ) is the estimated BOLD response, *X* captures the confounding effects, usually defined as a low-order discrete cosine that models low-frequency response drifts, with unknown coefficient β and ε is the error (Friston et al., 2003).

When using this formulation, noise and parameter priors are assumed Gaussian. The assumption is adopted to ease computation rather than to reflect biology (Lohmann et al., 2012). With this model definition in mind, we choose the model structure of classical DCM as:


$$\begin{array}{l} \frac{dz}{dt}=\underset{\text{A}}{\underset{︸}{\left[\begin{array}{ccc} {}* & * & *\\ {}* & * & *\\ {}* & * & * \end{array}\right]}}z+\underset{{\text{B}}_{1}}{\underset{︸}{\left[\begin{array}{ccc} {}* & * & *\\ {}* & * & *\\ {}* & * & * \end{array}\right]}}u_{1}z+\underset{{\text{B}}_{\text{2}}}{\underset{︸}{\left[\begin{array}{ccc} {}* & * & *\\ {}* & * & *\\ {}* & * & * \end{array}\right]}}u_{2}z+\text{B}\left[\begin{array}{c} u_{1}\\ u_{2} \end{array}\right],\\ \text{where B}=\left[\begin{array}{cc} {}* & *\\ 0 & 0\\ 0 & 0 \end{array}\right]\text{ or B}=\left[\begin{array}{cc} 0 & 0\\ {}* & *\\ 0 & 0 \end{array}\right]\text{ or B}=\left[\begin{array}{cc} 0 & 0\\ 0 & 0\\ {}* & * \end{array}\right], \end{array}$$

where * denotes the parameter which needs to be identified. Here, all nodes are assumed to be connected with *A* matrix having all ones. This assumption is supported by previous studies on the connectivity of these brain regions (Fastenrath et al., 2014; Ćurcić Blake et al., 2012; Gagnepain et al., 2017; Nawa and Ando, 2019; Smith et al., 2006). Most of these studies found a bidirectional connection between the nodes which matches our assumptions for this matrix.

For the *B*_*i*_ matrices, we allow both inputs (*u*_1_, *u*_2_) to modulate all couplings, so differences between emotional and neutral inputs are expressed in the degree to which they modulate each connection. A similar assumption for *B*_*i*_ matrices have been imposed in another study on emotional associative memory (Ćurcić Blake et al., 2012).

In the case of matrix *B*, it is unclear which brain regions receive a direct input. However, related studies (Fastenrath et al., 2014; Ćurcić Blake et al., 2012; Gagnepain et al., 2017; Nawa and Ando, 2019; Smith et al., 2006) found that the input acts directly on only one node which then influences the other nodes. Therefore, three different matrices are proposed, with inputs given solely to either Amy, Hip, or OFC.

#### 2.3.2 Stochastic DCM

Deterministic variations of DCM omit the random firing of neurons and variations in transmissions between neurons due to the stochasticity at the cellular and molecular level (Destexhe and Rudolph-Lilith, 2012; Guo et al., 2018; Stein et al., 2005; Faisal et al., 2008). To account for these, stochastic DCM includes an additive term to model neuronal noise (Li et al., 2011; Friston et al., 2011; Daunizeau et al., 2012). The resulting neuronal model, with ϖ as the noise, becomes:


$$\begin{array}{l} ż=Az+\underset{i}{\sum }u_{i}B_{i}z+Bu+\varpi \end{array}$$ (1)

The final model is then obtained by including the same hemodynamic model as the other DCM formulations. In the end, the stochastic DCM includes two noise terms: the measurement noise ε which is common for all types of DCM utilized in this paper, and the neuronal noise ϖ introduced in the stochastic DCM. The node connections of Section 2.3.1 are kept for the stochastic DCM as well, yielding three models.

## 3 Results

This section first presents the result of model comparisons among four model structures. We show that the stochastic DCM performed best. Thereafter, we discuss the choices of external inputs, i.e., which of the three brain regions receives the external input per condition, and provide a comparison between the model output and the measured data. Next, we explore the system properties, stability and controllability, of the obtained models in order to validate them from a dynamical systems' perspective.

### 3.1 Model selection

This research aims to identify a single best-fitting DCM for each condition (NN, NE, EE) that explains the data across participants. To this end, we first fitted every model of the model space to each participant's data for each of the 65 participants. By using four different model structures of DCM, we have examined 18 models to be evaluated: 3 classical DCM, 9 nonlinear DCM, 3 two-state DCM, and 3 stochastic DCM.

DCM models were estimated using variational Bayesian inference, which depends highly on prior specification. For all models, we adopted the default SPM12 priors without modification. After obtaining the subject-level models, we performed Bayesian Model Selection (BMS) separately for each condition (NN, NE, and EE). Model comparison used log model evidence. We adopted fixed-effects BMS, which assumes a single best model for all participants corresponding to one condition given the identical task and conditions. Under this assumption, the winning model is common across participants, only the parameter estimates differ. Finally, we performed Bayesian Parameter Averaging (BPA) to obtain group-level parameter estimates for the winning model structure.

To quantify goodness of the fitting, we compute the coefficient of determination (*R*^2^) between the DCM-predicted time series and group-average measured data. The measure is used to compute fraction of variance in the measured time series that can be explained by the model, i.e.,


$$\begin{array}{l} R^{2}=1-\frac{\sum_{i}{\left(y_{i}-f_{i}\right)}^{2}}{\sum_{i}{\left(y_{i}-ȳ\right)}^{2}}, \end{array}$$

where *y*_*i*_ is the measured signal at time *i*, *f*_*i*_ is the model prediction and ȳ is the mean of the measured signal. Positive (*R*^2^) values closer to 1 indicate better fit, whereas negative values arise when the model performs worse than a mean-only (flat) fit. The results of this metric are displayed on Table 1. For classical, nonlinear and two-state models the (*R*^2^) values are negative or close to zero. In contrast, the stochastic DCM yields mostly positive (*R*^2^) with larger magnitudes, except for the OFC in the EE condition, and slightly in the NE condition. These results, together with the qualitative observations from the figures, indicate that the stochastic DCM provides the best overall account of the data.

**Table 1:** The R-squared between the response of the optimal average models and the average measured data, calculated per condition, per brain region, and per DCM variation.

		$R_{\text{Amy}}^{2}$	$R_{\text{Hip}}^{2}$	$R_{\text{OFC}}^{2}$
Classical DCM	Condition NN	–6.7550	–7.8580	–3.1585
	Condition NE	–2.9134	–2.0622	–1.5884
	Condition EE	–3.3053	–3.2494	–0.0100
Nonlinear DCM	Condition NN	–0.0411	–0.8667	–0.2948
	Condition NE	–0.0422	–0.0120	–0.0272
	Condition EE	0.0075	0.0154	0.0005
Two-State DCM	Condition NN	–0.0411	–0.8667	-0.2948
	Condition NE	0.0241	0.0065	0.0088
	Condition EE	–1.9679	–2.1009	–0.0840
Stochastic DCM	Condition NN	0.6493	0.7626	0.3101
	Condition NE	0.3889	0.2011	–0.0344
	Condition EE	0.4802	0.5599	–0.6269

### 3.2 Stochastic DCM model: structure and accuracy

The stochastic DCM provided the best overall fit to the data, so we focus on this variant and examine its model structure, estimated parameters, state evolution properties and network connectivity.

#### 3.2.1 Model structure: Bayesian model selection

As outlined in Section 2.3.1, we evaluate three stochastic models that share the same structure for *A* and *B*_*i*_ matrices of neuronal state equations but differ in their *B* matrices to identify which of the three nodes, Amy, Hip, and OFC, the external input stimulates. Bayesian Model Selection (BMS) compares candidate models using the log model evidence. This value combines how well a model fits the data with how simple it is, by penalizing deviations from priors. The log evidence values for the stochastic DCM models are reported in Table 2. It is easier to evaluate these values on a scale relative to the lowest value. The relative log evidence values are obtained by ME_relative, model i_ = ME_absolute, model i_−min{ME_absolute_}. A relative log evidence of 3 or more is often considered as a strong indication for that model to be the optimal one. For each of the conditions, one of the models presents a relative log evidence of at least 3 compared to the other two models for that condition. These models are:

- Condition NN: Model 1 - input to Amy
- Condition NE: Model 3 - input to OFC
- Condition EE: Model 2 - input to Hip

**Table 2:** Absolute log model evidence values and posterior model probabilities obtained during Bayesian Model Selection (BMS).

Model	NN/NE/EE		
	NN	NE	EE
Log model evidence			
Model 1: Input to Amy	–0.0002	–32.0000	–11.4785
Model 2: Input to Hip	–32.0002	–32.0000	–0.0242
Model 3: Input to OFC	–8.5119	0.0000	–3.7351
Posterior probability			
Model 1: Input to Amy	0.9998	0.0000	0.0000
Model 2: Input to Hip	0.0000	0.0000	0.9760
Model 3: Input to OFC	0.0002	1.0000	0.0240

Looking across the three conditions, the log model evidence is highest for the NE condition. For the EE condition, model comparison is less decisive, as the difference in log evidence between Models 2 and 3 is only slightly above 3, indicating moderate evidence in favor of the winning model. Posterior probabilities, computed separately for each condition, nevertheless indicate a clear preference for a single model within each condition.

#### 3.2.2 Model accuracy

**Figure 4:**
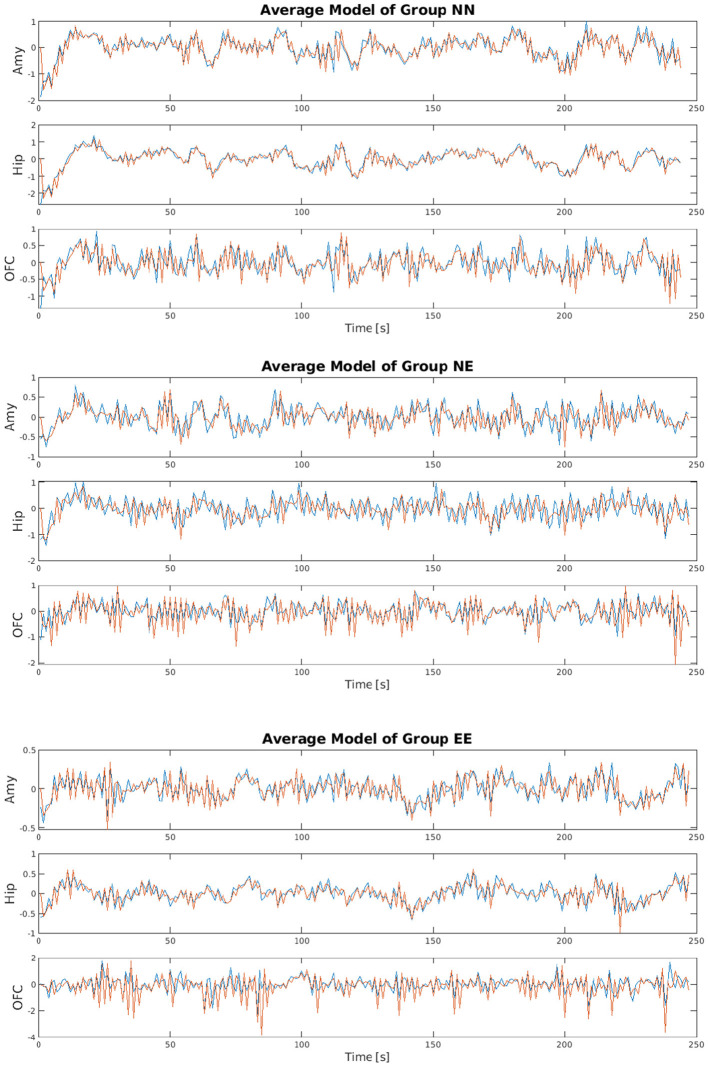
Stochastic DCM results for Amy, Hip, and OFC: blue line is the response of the average optimal model obtained for each condition; red line is the average signal for each condition.

Figure 4 shows the output of the group DCM model vs. the data. In DCM for fMRI, neuronal states typically start at their prior mean, commonly zero, and hemodynamic states at the steady states. Initial states are not usually estimated as free parameters. For the stochastic DCM, although the response also starts at zero, it quickly shifts to the level set by the data and then tracks it without a sustained offset.

To assess node-wise fit, we examined coefficients of determination (*R*^2^) for all candidate models rather than only the BMS winners. Table 3 reports these values separately for each node under each input mapping for each condition, NN, NE, and EE. Higher *R*^2^ values indicate better fit to the data. Comparing the models selected by BMS, *R*^2^ is highest in the NN condition. This can be explained by emotional images introducing more complex, less predictable neural dynamics that are harder to capture.

**Table 3:** R-squared values in each node (Amy, Hip, and OFC) for all average Stochastic DCM models.

	$R_{\text{Amy}}^{2}$	$R_{\text{Hip}}^{2}$	$R_{\text{OFC}}^{2}$
Condition NN			
Model 1: Input to Amy (optimal)	0.6493	0.7626	0.3101
Model 2: Input to Hip	0.4631	0.6996	0.0277
Model 3: Input to OFC	0.4632	0.6996	0.0278
Condition NE			
Model 1: Input to Amy	0.2719	0.0304	–0.5625
Model 2: Input to Hip	0.2717	0.0305	–0.5625
16-7.4,-13.5242ptModel 3: Input to OFC (optimal)	0.3890	0.2011	–0.0344
Condition EE			
Model 1: Input to Amy	0.4730	0.5534	–0.5530
Model 2: Input to Hip (optimal)	0.4802	0.5599	–0.6269
Model 3: Input to OFC	0.4730	0.5535	–0.5518

A second observation concerns the OFC in conditions NE, and particularly EE where the OFC *R*^2^ value is negative which means that the models perform worse than the mean-only reference. As shown on Table 4, subjects recorded in conditions NE and EE include fewer significant voxels in their OFC compared with the NN conditions which can depress the apparent fit. This pattern might have a few possible reasons, e.g., the OFC is challenging to image with fMRI due to susceptibility artifacts (Stenger, 2006); lower task engagement of the OFC under emotional conditions; or selection criteria for significant OFC voxel could be chosen slightly looser allowing inclusion of more OFC information in modeling. It is important to note that fewer voxels does not necessarily imply a minor role in the network. Coupling strength analysis in Section 3.3 show that they can still exert meaningful influences. However, future work should revisit the role of OFC.

**Table 4:** Number of significant voxels in the VOIs used to estimate group-optimal average models during BPA for all conditions.

	Number of significant voxels in BPA		
	Condition NN	Condition NE	Condition EE
Amy	177	729	217
Hip	347	212	421
OFC	509	69	7

Finally, the correlation between BMS and *R*^2^ measures varies by condition. In NN and NE, they agree for, that is the BMS-selected model also achieves the highest *R*^2^. However, in EE, *R*^2^ values are similar across model structures for each node. These results point to greater ambiguity and variability in the EE condition which can point to increased interference or noise.

### 3.3 Model validation: system properties

In this section, we use analytical tools, based on systems and control theory (see the Supplementary material) in order to assess the dynamic system properties of the selected models. These analyses allow us to rely on the inferred system properties for subsequent investigations, as well as to assess fundamental stability characteristics relevant to biological systems, such as bounded-input–bounded-response behavior. It is worth mentioning that the model we identified is composed of a deterministic part with an additive stochastic component. In stochastic DCM, process and observation noise are modeled as zero-mean Gaussian with bounded statistical properties, e.g., finite moments. Under these assumptions, stability and convergence of the underlying deterministic system constitute necessary conditions for the boundedness of the stochastic system. In our analysis, we focus on necessary conditions for mean-square stability of the stochastic DCM. In particular, the deterministic counterpart must be asymptotically stable in the absence of input, i.e., u = 0 (Ryashko and Schurz, 1997; Willems, 1973). For the case of non-zero but bounded inputs, piecewise constant in our setting, we adopt the notion of input-to-state stability (Sontag, 2008), whereby bounded inputs give rise to bounded system responses. Consequently, our analysis focuses on the deterministic component of the model. Controllability was evaluated solely to characterize the dynamical properties of this deterministic system. Morover, although the input varies over time, we approximate it as piecewise constant to facilitate interpretation. Stability was therefore analyzed within each interval under constant input.

#### 3.3.1 Stability: verification of the unforced model

It is not biologically plausible for neuronal activity to diverge exponentially to infinity. This implies that dynamical models of brain activity should exhibit stability. Consider deterministic counterpart of the identified model in Equation 1, i.e.,


$$\begin{array}{l} ż=Az+\overset{2}{\underset{i=1}{\sum }}u_{i}B_{i}z+Bu, \end{array}$$

where *u*_*i*_ ∈ {*u*_1_, *u*_2_} such that *u*_1_ corresponds to the input of the first association of each of the conditions NN, NE, or EE, and *u*_2_ to the second one. We verify the stability of the unforced, i.e., *u*_*i*_ = 0, and forced network dynamics. In what follows, we show that the unforced system corresponding to each of the conditions, NN, NE, and EE, are asymptotically stable, meaning that in the absence of excitation or input, deterministic neuronal activity will naturally decay over time. Property 1. The system matrix *A* corresponding to the model of each of the conditions NN, NE, and EE is asymptotically stable.

The above property verifies the validity of the identified unforced systems. We note that the values of elements of *A* matrices across the three conditions are close to each other. In order to provide a visible relation, below an average matrix is computed and sketched. The average couplings are calculated as,


$$\text{avg}\left(a_{i,j}\right)=\frac{\max a_{i,j}+\min a_{i,j}}{2},\;\text{var}\left(a_{i,j}\right)=\frac{\max a_{i,j}-\min a_{i,j}}{2}.$$

**Figure 5:**
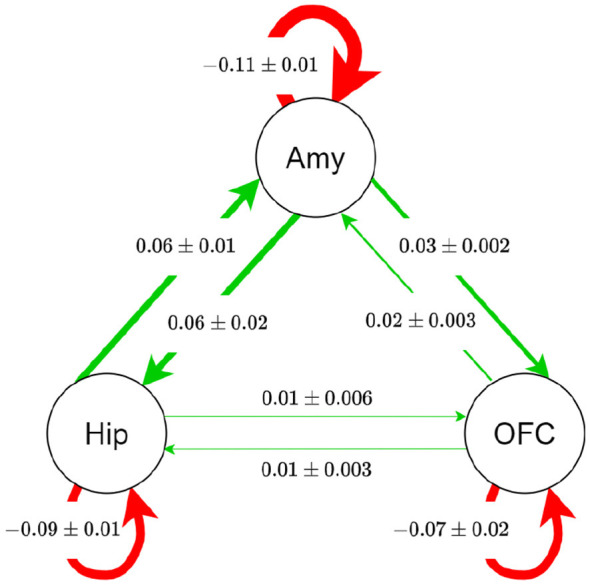
Values of elements of matrix *A* across the conditions NN, NE, and EE.

These coupling weights are illustrated on Figure 5. In this diagram, the line thickness defines the relative connection strength and the line colors indicate the sign with green as positive and red as negative. It is noteworthy that each of the directional couplings between two nodes has a comparable magnitude, representing an undirected graph. However, the pairs differ notably from one another, with Amy-Hip coupling being the strongest connection, and Hip-OFC the weakest. Finally, the largest couplings are associated with self-loops, particularly for the amygdala, indicating strong self-inhibition.

#### 3.3.2 Stability and controllability: verification of the bilinear model

Let us now consider the forced bilinear model. We now verify stability and controllability of the identified model. Verification of these properties allows us to infer that the identified model satisfies the expected dynamical properties, hence, it can be trusted in further analysis, for instance, perturbation analysis.

We first verify controllability. A system is controllable if its states can be driven to desired values via appropriate inputs, which in this context corresponds to the ability to modulate neural activity through changes in coupling strengths. Controllability was assessed using the rank condition of the controllability matrix (Åström and Murray, 2021) for each model across the three conditions. In all cases, the rank of the controllability matrix equals the system dimension (three), indicating that all models are controllable.

The dynamics of the forced model requires stability analysis with respect to its input. We verify the Input-State Stability (ISS) of the system (Sontag, 2008). We first do the analysis for the overall system considering |*u*_1_| ≤ 1, |*u*_2_| ≤ 1. Note that based on the experiment, the inputs *u*_1_ and *u*_2_ are not simultaneously present. Therefore, the model in Equation 1 can be described as ż = *Az*+*B*_*i*_*u*_*i*_*z*+*Bu*_*i*_, *i* ∈ {1, 2}. Property 2. The bilinear model ż = *Az*+*B*_*i*_*u*_*i*_*z*+*Bu*_*i*_, *i* ∈ {1, 2} is controllable. It is also input-to-state stable for all *u*_*i*_:|*u*_*i*_| ≤ 0.7.

Although the original unforced dynamics are asymptotically stable, input levels affect the stability when the system experiences external stimulation. We note that in the simulation results, *u*_*i*_ = 1 is used in the stochastic model which is switching between unforced and forced modes with two inputs. Above, we have characterized the condition under which the deterministic counterpart of the identified models are ISS in all conditions and for all admissible inputs. This condition is more conservative than the simulation where the inputs are applied for a limited duration, the model is subject to noise, hence, a loss of asymptotic boundedness does not have a visible impact in simulation. For the following connectivity analysis, we set *u*_*i*_ = 0.7 for mathematical consistency.

### 3.4 Analysis: network connectivity and coordination

After model validation, we perform connectivity analysis on the verified models to reveal the inter-regional mechanisms of emotional information encoding. Consider the dynamics of the network in the presence of one of the visual stimuli, we have,


$$ż=Az+B_{i}u_{i}z+Bu_{i},\;i\in \left\{1,2\right\}.$$

Let us denote the system equilibrium, i.e., the value of *x* for which ẋ = 0 holds, by x^*^. Define the error variable *e* = *x*−x^*^. We obtain,


$$\begin{array}{l} ė=A_{i}^{\prime }e+B_{i}u_{i}e,\;i\in \left\{1,2\right\}. \end{array}$$

As the inputs directly correspond to the stimulation by images, the model can be thought of a switched system that switches between an unforced and a forced system, with two forced modes *u*_1_ or *u*_2_, every 2.5*s*. Merging the intrinsic dynamics represented by matrix *A* with the effect of the extrinsic input, we can define the system matrix $A_{i}^{\prime }$ in (7) as


$$\begin{array}{l} A_{1}^{\prime }=A+B_{1}u_{1},\\ A_{2}^{\prime }=A+B_{2}u_{2}, \end{array}$$

where $A_{1}^{\prime }$ captures the dynamics when image “B” is shown (*u*_1_ = 1, *u*_2_ = 0) for 2.5*s*, and $A_{2}^{\prime }$ for image “C” (*u*_1_ = 0, *u*_2_ = 1). The elements of *A*′ matrices are provided in the Supplementary material.

Now, we have two linear systems, each corresponding to one of the inputs, and our purpose is to analyze the network connectivity and functional synchronization corresponding to each node. To this aim, we benefit from graph theory tools as reviewed in the Supplementary material. Let us define the graph Laplacian matrix as follows


$$\begin{array}{l} L=D_{\text{in}}-Ã, \end{array}$$

where $Ã\in \left\{A,A_{1}^{\prime },A_{2}^{\prime }\right\}$ is the adjacency matrix of the network and *D*_*in*_ is the diagonal node in-degree matrix. As known, the Laplacian algebraic connectivity λ_2_ characterizes the network's coordination: larger λ_2_ implies stronger coupling and faster consensus between neuronal activities. The network dynamics switches between input modes, and as shown in Olfati-Saber et al. (2007), the convergence rate is bounded by the λ_2_ values of the constituent graphs considering the switching schedule. We therefore examine λ_2_ for *A* (baseline), *A*_1_ (first cue), and *A*_2_ (second cue) as reported in Table 5.

**Table 5:** Algebraic connectivity λ_2_ in graphs and subgraphs for all conditions when no input is presented (adjacency matrix A), when *u*_1_ is active (adjacency matrix ${\text{A}}_{1}^{\prime }$), and when *u*_2_ is active (adjacency matrix ${\text{A}}_{2}^{\prime }$).

λ_**2**_ **of Graphs/subgraphs**				
	Amy→Hip	Amy←OFC	Hip←OFC	Entire graph
Condition NN				
A	0.1046	0.0461	0.0128	0.0411
${\text{A}}_{1}^{\prime }$	0.1077	0.0504	0.0123	0.0200
${\text{A}}_{2}^{\prime }$	0.1013	0.0214	0.0107	0.0227
μ(λ_2_)	0.1045	0.0393	0.0119	0.0279
Condition NE				
A	0.1457	0.0512	0.0187	0.0552
${\text{A}}_{1}^{\prime }$	0.1629	0.0666	0.0381	0.0832
${\text{A}}_{2}^{\prime }$	0.1456	0.0389	0.0189	0.0501
μ(λ_2_)	0.1514	0.0522	0.0252	0.0628
Condition EE				
A	0.1023	0.0449	0.0406	0.0620
${\text{A}}_{1}^{\prime }$	0.0930	0.0293	0.0284	0.0406
${\text{A}}_{2}^{\prime }$	0.1430	0.0638	0.0845	0.0976
μ(λ_2_)	0.1128	0.0460	0.0512	0.0667

The arrows show the dominant direction of connectivity between each two pair in each condition. The mean connectivity per condition and each two regions is denoted by μ(λ_2_).

**Remark:** We note that the Laplacian for signed graphs Kunegis et al. (2010) is most appropriate when effective connectivity includes both positive and negative couplings. In this work, however, we compute the classical graph Laplacian, whose second smallest eigenvalue corresponds to the algebraic connectivity. This choice is justified because nearly all edge weights are positive, and our focus is on changes in interregional coupling strength across conditions as inputs vary. In the NN condition (with input *u*_1_), two couplings are slightly negative but remain close to zero. Moreover, although the underlying graph is weighted and the resulting Laplacians are not strictly symmetric, our numerical computations in all cases produced matrices that satisfy the standard properties of Laplacians of connected undirected graphs (see the Supplementary materials).

The connectivity values (λ_2_) of the whole graph indicate that connectivity in the NN condition remains unchanged with alternating inputs. In the NE and EE conditions, connectivity differs between emotional and neutral image presentations. Relative to the unforced (baseline) network, NE shows an initial increase followed by a decrease in connectivity strength, whereas EE shows an initial decrease followed by an increase. Averaged across *u*_1_ and *u*_2_, both NE and EE exhibit higher overall network connectivity than baseline, with the increase being slightly larger for the EE condition.

**Figure 6:**
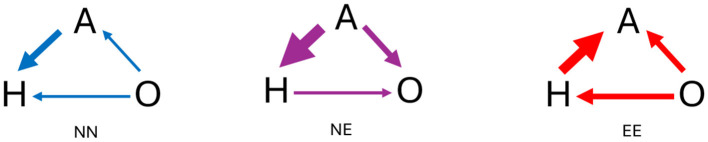
Dominant direction of connectivity in network corresponding to three conditions NN, NE, and EE.

In addition to the whole network analysis, we also study the sub-networks. Across all conditions, the λ_2_ values of the Amy-Hip sub-network are highest, indicating the strongest state coordination, e.g., consensus and synchronization, with the highest speed compared with Hip-OFC and Amy-OFC sub-networks. The highest values for Amy-Hip connectivity correspond to the NE condition, while in the EE there is increased connectivity compared to the NN condition. This indicates that the introduction of emotions leads to stronger interactions between the amygdala and the hippocampus. In contrast, Hip-OFC coupling possesses the lowest λ_2_ values, suggesting weaker and less robust coordination, except for condition EE. We shall note that the accuracy of the simulated model output for OFC vs. its corresponding data in condition EE is subject to error indicated by a negative *R*^2^ value in Table 3. The latter may influence the inferred couplings between OFC and the two other regions in the condition EE. Nonetheless, our analysis stays coherent across the other conditions allowing hypothesizing the weaker connectivity of Hip-OFC. Figure 6 shows the dominant direction of influence per condition, based on Table 5, together with the intensity of the flow, i.e., connectivity, across the three conditions. Other couplings are omitted for the sake of clarity.

### 3.5 Implications of degree of network connectivity for memory integration and recall

The algebraic connectivity, λ_2_, provides a graph-theoretical measure of how tightly or loosely coupled a network is, reflecting the overall coherence of interactions among its nodes. Higher λ_2_ values indicate stronger coordination of activity across the network, suggesting that regions are more integrated. For instance, an increase in λ_2_ within the Amygdala–Hippocampus sub-network implies that the activity of these two regions becomes more strongly interdependent, such that fluctuations in one region are more closely mirrored by the other. Our interpretation linking higher λ_2_ to memory enhancement, as well as integration, is supported by findings in Zhu et al. (2023) based on the same dataset, where stronger amygdala–hippocampal coupling and improved memory performance were both observed in the NE condition. Accordingly, our connectivity results are consistent with this interpretation and point toward a potential predictive relationship with subsequent recall performance, as also discussed in Zhu et al. (2023). While the behavioral correlates associated with the orbitofrontal cortex (OFC) remain largely unexplored, and were not explicitly tested in Zhu et al. (2023), our analysis suggests that in the EE condition the coupling between the OFC and the hippocampus is stronger than in the other two conditions. Moreover, the model appears more sensitive to increases in the second emotional valence. Together, these findings suggest that the two emotional components may interact in a way that leads to interference effects, such that memory integration and recall in the EE condition are weaker compared to the NE condition.

### 3.6 Sensitivity of the model and prediction

We have analyzed network connectivity per condition using graph-theory measure, i.e., the algebraic connectivity, and showed that introducing emotional arousal increased overall network connectivity, particularly hippocampus–amygdala coupling. The latter is observed strongest in the NE condition. An interesting question is to determine the effects of increasing the intensity of neutral and emotional cues. We verify this question by varying the input signals and test system properties and network connectivity. In other words, we verify changes in system's stability and the network's algebraic connectivity by increasing the values of *u*_1_ and *u*_2_. We now provide a group-wise analysis by assuming perturbations of the input signals.

Condition NN: the upper bound of *u*_1_ for guaranteeing the stability of $A_{1}^{\prime }$ is even smaller than the threshold for other inputs. For *u*_2_, the input can be increased two times to keep the network stability, however, matrix $A_{2}^{\prime }$ has mixed positive and negative diagonal and off-diagonal elements which influences network coordination.

Condition NE: considering matrix *B*_1_ corresponding to *u*_1_, we can increase the input by 10 times before $A_{1}^{\prime }$ loses stability. However, the structure of the corresponding Laplacian matrix changes for *u*_1_≥6.5. Thus, the network connectivity increases up to *u*_1_ = 6.5. Considering *u*_2_, a slight increase in the value of *u*_2_ leads to instability of $A_{2}^{\prime }$.

Condition EE: When *u*_2_ is increased, $A_{2}^{\prime }$ becomes unstable. In contrast, increasing *u*_1_ by three fold keeps $A_{1}^{\prime }$ stable, although measured connectivity declines as input increases. Thus, compared with NE, the EE model is less robust. However, its algebraic connectivity increases by decreasing *u*_1_, and similarly by increasing *u*_2_.

Our model then predicts that in the NE condition, making the first image more neutral, as a manner of increasing the intensity of *u*_1_, increases the measured connectivity, hence, we infer a more enhanced memory. Also, in the EE condition, if the first emotional trigger is less intense compared with the second one, the encoding is enhanced. Looking at both of the conditions, the result is consistent: the more the first trigger carries less emotional valance compared with the second one, the greater level of integrated activities in hippocampus-amygdala occur leading to improved associated memory.

## 4 Discussion

Here, we discuss the main objectives of the article, modeling and mechanistic understanding by analysis, by shedding a light on the obtained results and discuss limitations and future perspectives.

We have explored several dynamic models and found that a stochastic bilinear state-space model is the best candidate for emotional memory encoding in all three conditions, neutral-neutral, neutral-emotional and emotional-emotional. We have used control theory tools, such as input-state stability, to validate the deterministic counterpart of the model. We then measured graph algebraic connectivity of the networks which is an indicator of the network connectivity and functional coordination among the amygdala, hippocampus, and OFC. Our results confirm the prior findings in Zhu et al. (2023) that memory was enhanced in the neutral-emotional condition, as we showed the highest increase of the hippocampus–amygdala coupling in this condition compared with the other two. It is worth noting that in the NE condition, the OFC–hippocampus coupling is the weakest compared with the couplings between the amygdala and the other two regions. Furthermore, analyzing the input-state stability of the model of emotional–emotional condition, we discussed that the coupling of hippocampus and amygdala increases when the first image's valence is substantially less negative rated than the second image, but decrease otherwise. This pattern mirrors the neutral–emotional condition, where the first image is emotionally neutral compared with the second. Moreover, our effective connectivity analysis suggested that the amygdala predominantly influences the other two regions in the neutral–emotional condition. Our analysis also suggests that the OFC plays a dominant role in the other two conditions although the low number of significant OFC voxels, particularly in the EE condition shall be noted in this conclusion. However, we shall notice that the hippocampus-amygdala dynamics in our work is still reliable as (1) the *R*^2^ values for all possible inputs in the EE condition lead to closely equal hippocampus-amygdala dynamics; (2) the strength of connection from OFC to hippocampus or amygdala is weaker compared with the hippocampus-amygdala coupling, (3) the results in Zhu et al. (2023) performing data analysis in the absence of OFC confirms a similar dynamics for hippocampus-amygdala in the EE condition. In what follows we further elaborate on the model, analysis, limitations and perspectives.

### 4.1 Stochastic bilinear network model

As discussed in the modeling part, stochastic bilinear model served as the best fit to the experimental data of emotional encoding. In fact, stochastic DCM includes nonlinearities via the input-dependent bilinear term, and in addition, it incorporates the unmodeled and noisy elements via the stochastic component. One of the most crucial differences between the stochastic DCM and the other DCM variations examined in this work is the use of generalized coordinates which allows tracking several time derivatives of each estimated parameter, as opposed to considering only point estimates. Consequently, much more information about the shape of the function is considered, allowing the model to correctly represent vastly more complex signals.

Moreover, experimental evidence supports including stochastic uncertainties in our modeling. Neural noise tends to increase when the environment diverges from a person's predictions (Faisal et al., 2008). In our task, images were shown rapidly and evoked highly subjective emotional responses; each valence was presented for about 8 seconds, a duration comparable to the hemodynamic response time. We note that fMRI estimates neural activity indirectly by measuring the BOLD signals which comparably posses slower dynamics. Therefore, increasing deterministic nonlinear components to capture changing dynamics is not appropriate, as reflected in our modeling choices. Stochastic DCMs accommodate these temporal fluctuations by linking fast neuronal dynamics to the slower BOLD signal.

### 4.2 Analysis for revealing mechanisms

We used graph theory and stability theory tools in order to analyze the modeled dynamics and reveal its mechanisms. Our analysis on network coordination show dependency on input context, that is, the order of the emotional valence of the images, i.e., neutral or emotional, and their intensity shape the associative memory encoding dynamics. We showed that across conditions the amygdala-hippocampus pair consistently exhibits higher algebraic connectivity than other pairs. This result supports the view that the limbic amygdala–hippocampus loop anchors emotional associative encoding. We observed that the algebraic connectivity of the whole graph is higher in conditions containing emotional cues, i.e., neutral-emotional and emotional-emotional, consistent with emotion-induced encoding. This increase is not persistent and differs in nature between these two conditions. In neutral-emotional, a more intense neutrality before an emotional cue increases hippocampus-amygdala connectivity, which we interpret as greater enhanced memory. In emotional-emotional, a milder first, or a stronger second, emotional valence is associated with higher network connectivity and integration; increasing the first image's emotional valence appears to reduce integration. Both findings align and together suggest that the second emotional information should be more intense to improve information integration via Amy-Hip coupling. In addition, our modeling approach infers effective connectivity, i.e., the neuronal activity of which of the three brain regions has a greater influence on the two others. Our results indicate that, in the neutral-emotional condition, the amygdala is the major influencer in the network.

Overall, we have shown that a combined connectivity and stability analysis allow us to determine how to tune the level of emotional valence of the cues to improve or disrupt information encoding.

### 4.3 Biological contextualization of the results

Our findings can be interpreted within established neurobiological frameworks of emotional memory, particularly *emotional tagging* models, which posit that arousal-related amygdala activity retroactively enhances the consolidation of associated representations in the hippocampus, e.g., McGaugh (2004) and Richter-Levin and Akirav (2003). The observed increase in hippocampus–amygdala coupling in the neutral–emotional condition is consistent with evidence that the amygdala modulates hippocampal plasticity in an arousal-dependent manner, thereby prioritizing behaviorally relevant information (Fastenrath et al., 2014; Dolcos et al., 2004). Moreover, the directionality of information flow we identify, particularly the amygdala's leading role in the NE condition, aligns with models in which the amygdala acts as a relevance detector that gates hippocampal encoding processes (Phelps, 2004; Roozendaal and McGaugh, 2011). The differential effects of stimulus order and intensity observed here further resonate with literature showing that excessive or competing emotional arousal can impair associative binding, likely due to resource competition or interference within medial temporal lobe circuits (Bisby and Burgess, 2013; Mather and Sutherland, 2011). Together, our results extend this framework by suggesting that not only the presence but also the temporal structure of emotional signals shapes amygdala–hippocampal dynamics and, consequently, the success of associative memory integration.

### 4.4 Limitations and perspectives

As a known limitation of Dynamic Causal Modeling (DCM), exploring large model spaces is computationally demanding. Nevertheless, the full capacity of DCM was not exhausted in the present study, and additional regions may be considered in future extensions.

Our results delineate a principled model space that can guide future data-driven investigations of emotion–memory interactions across modeling frameworks. We adopted a hypothesis-driven approach to examine the core circuitry underlying emotional enhancement of memory, focusing on interactions between the amygdala, hippocampus, and orbitofrontal cortex (OFC). These regions are well established in the modulatory influence of emotion on memory encoding and consolidation (McGaugh, 2004; Phelps, 2004). We restricted the region-of-interest (ROI) set to maximize interpretability and sensitivity to emotional modulation. Although posterior regions, e.g., angular gyrus, precuneus, posterior cingulate cortex, ventral visual areas, are key components of large-scale memory networks, e.g., Ranganath and Ritchey (2012), their inclusion would substantially increase model complexity and extend beyond the scope of the present work. Our ROI selection was further guided by prior work using the same dataset (Zhu et al., 2023), which focused on the left amygdala and the hippocampus. Consistent with this and our hypothesis-driven framework, we restricted analyses to left-lateralized ROIs. This restriction reduces model complexity and the number of hidden states, facilitating DCM estimation (Zeidman et al., 2019). Moreover, interhemispheric connectivity is dynamically modulated and may increase variability in effective connectivity estimates (Grefkes et al., 2008; Almgren et al., 2018). The resulting models captured the neuronal dynamics of interest, supporting the adequacy of this circuit for our research questions.

Although emotional processing is often described as right-lateralized, evidence for consistent hemispheric asymmetries is mixed. Studies using similar paradigms report comparable effects across hemispheres, and meta-analyses suggest that amygdala lateralization depends on task and stimulus characteristics rather than reflecting a general principle (Baas et al., 2004). This study was not designed to assess hemispheric lateralization. The relaxation of the unilateral restriction to bilateral and larger-scale network architectures serve as worth-investigating future avenues.

Additionally, low numbers of informative voxels in the OFC reduced predictive accuracy, suggesting that more detailed investigation of the OFC's role in the network—potentially with larger datasets—could add to the obtained knowledge.

In this work, we focused on the group-level modeling, and used the fixed-effect BMS for this aim. Our experiment involved 70 participants in total, a sample size considered sufficient for stable group-level inference. The participants were clustered to three conditions, creating relative homogeneity within each group and mitigating inter-individual variability. The outliers, those who did not complete the task or fell asleep during recording, have been excluded from the data set (Zhu et al., 2023). Independent modeling of each condition revealed the same optimal model type across participants, produced a similar correlation between the Hippocampus and Amygdala as reported in Zhu et al. (2023). Comparing group and individual-level models and quantifying their correspondence is an important question that should be addressed in future work.

Another key future step is to integrate the recall phase of the experiment into the connectivity analysis to verify how connectivity during encoding relates to subsequent recall or forgetting. Using the same dataset as in Zhu et al. (2023), our model similarly indicated stronger amygdala–hippocampus connectivity in the neutral-emotional (NE) condition. We have therefore hypothesized that higher hippocampus-amygdala algebraic connectivity (e.g., λ_2_) is associated with better recall performance. In particular, because input influences both connectivity and stability, future experiments that systematically manipulate emotional valence while measuring its effects on both encoding and recall would be valuable for improving our understanding of these mechanisms, as well as for testing the predictions proposed in this study.

Finally, assessing the applicability of these results for designing regulatory mechanisms, such as precision-medicine approaches or stimulation techniques, and examining implications for learning and education are important directions for future research.

## 5 Conclusion

We studied the network dynamics composed of the hippocampus, amygdala, and orbitofrontal cortex in an fMRI emotional associative-memory task. Participants viewed image pairs that were neutral–neutral, neutral–emotional, or emotional–emotional. Using the dynamic causal modeling framework, we showed that a stochastic bilinear state-space model best describes the neuronal dynamics in each condition. Furthermore, we analyzed the network dynamics of each condition using tools from graph theory and stability theory and discussed the differences in strength and direction of couplings, connectivity and stability of each of these networks. Our results confirmed the prior findings that introducing emotional stimuli increases hippocampus–amygdala coupling in the neutral–emotional condition. In addition, we predicted that in the emotional–emotional condition, amygdala-hippocampus connectivity further increased when the first image's valence was substantially less negative than the second's, but decreased otherwise. We inferred that the highest overal network connectivity in the emotional-emotional condition may indicate the interfering effects. Our analyses revealed the effective connectivity and suggested that the neuronal activity of amygdala may influence the other two regions in the neutral–emotional condition. We provided an extensive discussions about our findings and suggested future directions. Particularly, we pointed out to the utility of control theory and graph theory tools in enhancing the mechanistic understanding of neural dynamics and their potentials to inform the development of future technological or pharmacological approaches targeting regulatory mechanisms.

## Data Availability

The experimental training data that support the findings of this research were provided by the Radboud Data Repository (https://data.ru.nl/). The project was named ‘Emotional Modulation of the Interaction Between Related Memories with Functional MRI' in the repository (https://doi.org/10.34973/esn0-yf75).
